# Strain-dependent emergence of aminoglycoside resistance in *Escherichia coli* biofilms

**DOI:** 10.1016/j.bioflm.2025.100273

**Published:** 2025-03-12

**Authors:** Raphaël Charron, Pierre Lemée, Antoine Huguet, Ornella Minlong, Marine Boulanger, Paméla Houée, Christophe Soumet, Romain Briandet, Arnaud Bridier

**Affiliations:** a Antibiotics, Biocides, Residues and Resistance Unit, Fougères Laboratory, Fougères, Anses, 35300, France; b Université Paris-Saclay, INRAE, AgroParisTech, Micalis Institute, 78350, Jouy-en-Josas, France

**Keywords:** Biofilms, Antibiotics, Resistance, Adaptive evolution, Aminoglycoside

## Abstract

In most Earth environments, bacteria predominantly exist within surface-associated communities known as biofilms, where they are embedded in an extracellular matrix. These collective structures play a critical role in bacterial physiology and significantly shape their evolutionary trajectories, contributing to the development of antimicrobial resistance and enhancing bacterial resilience to treatments, with profound implications for public health. This study assessed the impact of the biofilm lifestyle on the emergence of resistance to gentamicin, an aminoglycoside antibiotic, in one laboratory reference strain and seven *Escherichia coli* isolates from food-processing environments. Throughout a one-month evolution experiment, we observed that certain strains showed a markedly higher emergence of gentamicin-resistant variants in biofilms than in planktonic states, with the emergence of stable variants being closely linked to biofilm maturation. Genomic and phenotypic analyses of gentamicin-resistant (GenR) variants uncovered varied adaptive strategies among the strains. GenR variants from two food-processing isolates (Ec709 and Ec478) displayed point mutations in genes associated with central carbon metabolism (*aceE*, *ygfZ*, …) and cell respiration (*atpG*, *cydA*, …), while retaining relative growth and colonization capacities. Conversely, GenR variants from the reference strain (Ec1655) adapted preferentially through large genomic deletions, including consistent loss of the peptide transporter gene *sbmA,* significantly altering cellular fitness. These findings highlight the complexity of adaptive evolution in biofilms and underscore the importance of investigating diverse strains to grasp the full spectrum of adaptation in natural bacterial populations.

## Highlights

• Biofilms promote strain-dependent emergence of gentamicin-resistant phenotypes.
• Gentamicin resistance correlates with biofilm maturation and metabolism slow-down.
• Genomic analysis reveals diverse adaptation strategies among strains.
• Large deletion confer high resistance but incur significant fitness cost compared to SNPs.

## 1 Introduction

The discovery of antibiotics revolutionized both human and veterinary medicine, saving millions of lives. Nevertheless, this success is now jeopardized by the spread of antibiotic-resistant bacteria. The misuse of antibiotics—through improper medical practices or inappropriate applications, such as their use as growth promoters in agriculture [1]—has accelerated the selection of bacteria capable of surviving these therapeutic agents. A diverse range of mechanisms that drive the emergence and selection of antibiotic resistance has been identified across various bacterial species, including pathogens [2,3].

In natural environments, bacteria predominantly inhabit surface-associated communal populations called biofilms, rather than the individual free-floating planktonic state typically studied in laboratories [4]. Within biofilms, microorganisms are spatially organized and embedded in an extracellular matrix composed of diverse molecules such as polysaccharides, lipids, extracellular DNA and proteins [5]. This collective lifestyle fosters the development of heterogeneous populations, potentially including antibiotic-tolerant subpopulations [6]. The extracellular matrix and resource consumption by bacteria at the biofilm's outer layers often restrict the diffusion of nutrients and oxygen to deeper layers [6], inducing bacterial adaptations to nutrient scarcity, such as slow- or non-growing states [7]. These physiological changes can enhance bacterial survival under antimicrobial stresses, as inactive metabolic pathways reduce vulnerability to antimicrobial toxicity [8]. The biofilm environment is however stressful for the cells, often triggering the activation of stress responses, such as the stringent and the SOS responses [9]. These responses often involve the use of error-prone polymerases, which facilitate rapid DNA repair, but also increase the likelihood of non-synonymous mutations, thereby promoting the emergence of resistance mutations [10]. Resistance mutations often come with fitness costs [11], but biofilms can act as a “protective cocoon” [9], enabling resistant bacteria to establish themselves and develop compensatory mutations, which mitigate these costs [12].

These unique biofilm-specific features make this lifestyle a powerful driver of bacterial evolution, promoting the emergence of antibiotic-resistant clones, even in the absence of external stresses. In this study, we explored how the biofilm lifestyle affects the emergence of gentamicin-resistant (GenR) bacteria. Gentamicin, an aminoglycoside antibiotic classified as critically important by the World Health Organization [13], serves as a valuable model for studying variants emergence within biofilms. Resistance to aminoglycosides arises through mutations in genes such as the elongation factor *fusA* [14,15], peptide transporters like *sbmA* [15], or genes involved in cell respiration pathways (e.g., *atpG*, *cydA*) [14,16] and other metabolic pathways (e.g., *aceE*) [17]. In this work, we assessed the biofilm profiles of eight *E. coli* strains, including isolates recently obtained from food-processing environments. Using experimental evolution, we investigated the influence of the biofilm lifestyle on the selection and emergence of GenR variants within a One Health framework.

## 2 Materials and methods

### 2.1 Bacterial strains

This study utilized eight *E. coli* strains, all of which were confirmed to be susceptible to gentamicin (MIC <0.5 mg/L). The MG1655 strain, hereafter referred to as Ec1655, served as a reference strain. Another strain, CIRMBP-0223 (Ec223), was obtained from the CIRM collection at INRAE and originates from a chick. The remaining six strains (Ec478; Ec694; Ec709; Ec723; Ec775; Ec956) were sourced from the National Reference Laboratory for Antimicrobial Resistance collection, housed at the ANSES laboratory in Fougères. These isolates were collected from the pork and poultry industries between 2014 and 2019. All bacterial strains were stored at −80 °C in cryotubes (Mast Group, Bootle, UK).

### 2.2 Biofilm structure analysis by Confocal Laser Scanning Microscopy (CLSM)

Bacterial strains were first streaked from cryotubes onto Tryptone Soy Agar (TSA) plates and incubated overnight at 37 °C. A single colony was then suspended in ten-fold diluted Tryptone Soy Broth (TSB 1/10) and incubated overnight at 37 °C. The following day, cultures were diluted to achieve a final OD_600 nm_ of 0.2. For biofilm formation, 10 μL of the diluted suspension was added to 190 μL of TSB 1/10 in a microplate well. The plate was incubated at 19 °C for 1 h, after which the medium was replaced with fresh TSB 1/10. Biofilms were subsequently incubated under static conditions at 19 °C for 72 h. After the incubation period, biofilms were stained with SYTO™9, a green fluorescent permeant nucleic acid stain (Invitrogen, USA), combined with either DeepRed Concanavalin A (Invitrogen, USA) (ConA) or red Wheat Germ Agglutinin (Invitrogen, USA) (WGA). Imaging was performed using a Leica HCS-SP8 CLSM at the INRAE MIMA2 Imaging Core Facility [18]. Fluorophores were excited at 488 nm (SYTO™9), 561 nm (WGA) and 633 nm (ConA), with emissions collected using photomultiplier tubes (PMT) at 500–550 nm (SYTO™9), 600–750 nm (WGA) and 650–750 nm (ConA). Biofilms were scanned using a 63x water objective lens (numerical aperture: 1.2), with an image resolution of 512x512 and a 1 μm Z-step. Two wells were imaged for each condition, with two fields acquired per well. 3D biofilm reconstructions were generated using Imaris 9.3.1 software (Bitplane, AG-Zürich, Switzerland). Quantitative biovolumes for ConA and WGA staining were extracted using BiofilmQ 0.2.2 [19]**.**

### 2.3 RT-q-PCR analysis of Ec1655 biofilms

Gene expression in Ec1655 biofilms was quantified over a one-month period. Genes were selected to represent key cellular functions, more precisely biofilm formation (due to its relevance in this model), stress responses (linked to bacterial recalcitrance and bacterial growth), transport (associated with resistance), and cell metabolism (closely related to gentamicin resistance). The medium was renewed daily with fresh TSB 1/10. Biofilms from eight wells were pooled at days 3 (the day after the initial 72-h incubation), 10, 17 and 31. Total RNA was extracted using the NucleoSpin RNA kit according to manufacturer's instructions (Macherey-Nagel, Hoerd, France) with some specific modifications. An enzymatic lysis step was introduced, involving incubation with 25 μL of a 1 mg/mL lysozyme, Tris 10 mM and EDTA 1 mM solution at 37 °C for 10 min. RNA concentration and purity were assessed using a Biospec-Nano spectrophotometer (Shimadzu, Marne la Vallée, France). Primer design, reverse transcription, qPCR, and analyses were performed as described in Reale et al. [20], with some modifications. Reverse transcription was carried out using 1.3 μg of total RNA, and qPCR reactions were performed with 2.6 ng cDNA. Primers were purchased from Sigma-Aldrich (Saint-Quentin Fallavier, France), with detailed primer sequences provided in Table S1. For normalization, the *hcat* gene was selected as the reference, as it exhibited stable expression, confirmed using NormFinder software. Three independent experiments were performed to ensure reproductibility.

### 2.4 Biofilm and planktonic experimental evolution

Biofilms were established as previously described, with eight replicates per strain. For planktonic cultures, 10 μL of bacterial suspensions (OD_600 nm_ = 0.2) were added to 190 μL of TSB 1/10, and incubated at 19 °C with shaking (200 rpm) for 72 h. At specific timepoints (days 3, 10, 17, 24 and 31), 5 μL of biofilm supernatants were plated on agar containing 10 mg/L gentamicin, a concentration identified in preliminary experiments as the lowest threshold differentiating wild-type and adapted phenotypes. Supernatants were subsequently removed and 40 μL of fresh 1/10 TSB were added. Biofilms were resuspended and 5 μL of the suspension was plated on the gentamicin-supplemented plates. In parallel, 5 μL of the planktonic plates were plated under the same conditions. Plates were incubated at 37 °C for 48 h. Medium was renewed by filling each biofilm well to a total volume of 200 μL. Planktonic cultures renewal was performed with a transfer of 10 μL of the old suspension in 190 μL of fresh TSB 1/10 medium. Plates were incubated at 19 °C (statically for biofilms, with 200 rpm for planktonic cultures). Medium was renewed every day, the biofilm supernatants were discarded and replaced with 200 μL of fresh TSB 1/10 medium. In parallel, 10 μL of the planktonic cultures were transferred in 190 μL of fresh TSB 1/10 medium.

### 2.5 Mutation stability assay

The stability of resistance phenotypes in variants was assessed following the protocol of Charron et al. [21]**.** Variants were streaked onto TSA plates and incubated at 37 °C for 24 h, with this process repeated four consecutive times. On the fifth passage, variants were plated onto TSA containing 10 mg/L gentamicin to confirm the retention of resistance.

### 2.6 Enterobacterial repetitive intergenic Consensus – PCR (ERIC-PCR)

ERIC-PCR was performed to verify that variants originated from the parental strain, following the method described in Charron et al. [21]**.** Briefly, DNA from both the parental and variant strains was extracted using the InstaGene kit (Bio-Rad, Marnes-la-Coquette, France). ERIC-1 (ATGTAAGCTCCTGGGGATTCAC) and ERIC-2 (AAGTAAGTGACTGGGGTGAGCG) primers were used to amplify specific bands with the GoTaq Flexi polymerase (Promega, Charbonnières-les-Bains, France) in a LightCycler 480 thermocycler (Roche Diagnostics, Meylan, France), with the following parameters: an initial denaturation at 95 °C for 2 min; followed by 30 cycles of 95 °C for 1 min, 54 °C for 1 min and 72 °C for 4 min. A final extension step was carried out at 72 °C for 8 min followed by an unlimited hold at 4 °C. The PCR products were analyzed by agarose gel electrophoresis to determine whether the variants originated from the parental strain.

### 2.7 DNA extraction, sequencing and genomic analyses

**Fig. 1:**
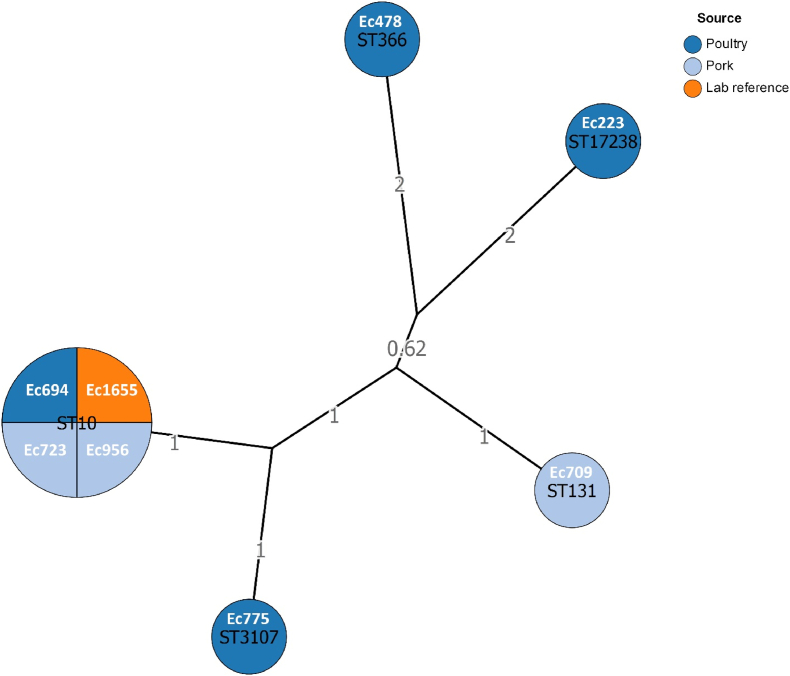
Grape tree network illustrating the MLST relationships among the 8 parental strains. The edges represent the allelic distances between the different *E. coli* sequence types. Each node is labeled with the strain name and the corresponding sequence type identified through MLST analysis.

Whole genome sequencing of all *E. coli* strains was performed using paired-end short-reads sequencing. Bacterial cultures were grown overnight on Thermo Scientific™ Tryptone Soya Agar with 5 % Sheep Blood plates (Thermo Fisher Scientific, PB5012A) at 37 °C. A single colony was inoculated into 5 mL of TSB 1/10 and incubated overnight at 37 °C. Total DNA was extracted using the Nucleospin Tissue kit (Macherey-Nagel, Düren, Germany), according to the manufacturer's instructions. DNA was quantified using the Qubit dsDNA BR HS assay kit with a Qubit 4 fluorometer (Thermo Fisher Scientific, Courtaboeuf, France), according to manufacturer's instructions. Illumina sequencing was performed at Institut du Cerveau et de la Moelle Epinière (Paris, France) on a Novaseq 6000 SP. Nextera XT kit was used to prepare Illumina libraries. Sequence data was processed as described in Lemée et al. [22]. Reads were trimmed using fastp (0.23.4) [23] and genomes assembled using Unicycler (v0.5.0) [24], with only contigs longer than 200 nt retained. Genomic annotation was carried out using Bakta (v1.8.2) [25]. Variant calling was conducted for every variant, with Snippy (v4.6.0) (https://github.com/tseemann/snippy), with the parental strain genome serving as the reference. Analysis pipelines are available on GitHub: https://github.com/AB2R/WGS_pipeline.git. Multi-locus sequence typing (MLST) was performed on each parental strain, using the Achtman scheme, which covers seven loci [26]. A Grapetree was generated using Enterobase [27,28], employing the neighbor-joining algorithm to visualize the relatedness of the strains (Fig. 1).

### 2.8 Minimal inhibitory concentration (MIC) determination for 15 antibiotics

The MIC for 15 different antibiotics was determined as previously described in Cuzin et al. [17]. Bacterial strains were streaked onto TSA plates containing 5 % Sheep blood and incubated overnight at 37 °C. The following day, colonies were suspended in 5 mL of sterile water to achieve a turbidity of 0.5 Mac Farland (≈10**^8^** CFU/mL). A 10 μL aliquot of this suspension was transferred into 11 mL of Mueller Hinton Broth (MHB). A 50 μL volume of the prepared MHB suspension was inoculated onto a standardized microplate (EUVSEC, Sensititre®, TREK Diagnostic Systems Ltd., Thermo Fisher Scientific, East Grinstead, UK) containing lyophilized antibiotics. Plates were incubated for 24 h at 35 °C, and MIC values were recorded the following day. MIC values were determined for each variant and parental strain, as the median value of three replicates.

### 2.9 Bacterial growth kinetics analysis

Growth kinetics of the bacterial strains were analyzed over an 18-h period. Bacterial strains were subcultured overnight in 1/10 TSB at 37 °C. The following day, cultures were adjusted to an OD_600nm_ of 0.2, and 10 μL of the suspension was transferred into 190 μL of 1/10 TSB to achieve a final OD_600nm_ of 0.01. Cultures were incubated at 20 °C (±1 °C) in a FLUOstar OPTIMA (BMG Labtech, Champigny-sur-Marne, France) for 18 h, with OD measurements taken every 30 min. Growth rates (μmax) were extracted using the MARS Data analysis software (BMG Labtech, Champigny-sur-Marne, France). Three biological replicates, each with two technical replicates, were performed, and individual data points are represented in the figure.

### 2.10 Quantification of biofilm production by crystal violet staining

Crystal violet staining of biofilms was performed following the method described by Merritt et al. [29]**.** Variants and wild-type strains were grown overnight at 37 °C in 5 mL of TSB 1/10. Biofilms were prepared as described in section 2. After 72 h of incubation, supernatants were removed, and wells were washed with 200 μL of a sterile NaCl solution (150 mM). Biofilms were stained with 125 μL of a 0.1 % crystal violet solution for 10 min. Excess stain was removed, and wells were washed twice with 200 μL of a sterile NaCl solution (150 mM). The bound crystal violet was solubilized by adding 200 μL of an ethanol (80 %)/acetone (20 %) solution to each well, followed by a 15 min incubation. The resulting supernatants were transferred to a new plate, and absorbance at 590 nm (OD_590nm_) was measured using a FLUOstar OPTIMA (BMG Labtech, Champigny-sur-Marne, France). Two independent biological replicates, each with three technical replicates, were performed, and individual datapoints are represented in the figure.

### 2.11 Statistical analysis

Statistical analyses were performed using Graphpad Prism software. The Kruskal-Wallis test, combined with Dunn's multiple comparison test, was used for comparisons of the parental strains (ConA and WGA biovolume values and Congo red fluorescence values), RT-q-PCR analysis (for each gene and sampling day), and comparisons of biofilms, supernatants and planktonic conditions (for each strain and sampling day). Growth parameters and crystal violet assays were compared using the Mann-Whitney test between variants and their parental strains. P values are presented as follows: ∗ = [0.05–0.01]; ∗∗ = [0.01–0.001]; ∗∗∗ = <0.001.

## 3 Results

### 3.1 Biofilms promote the emergence of stable GenR variants in Ec1655

**Fig. 2:**
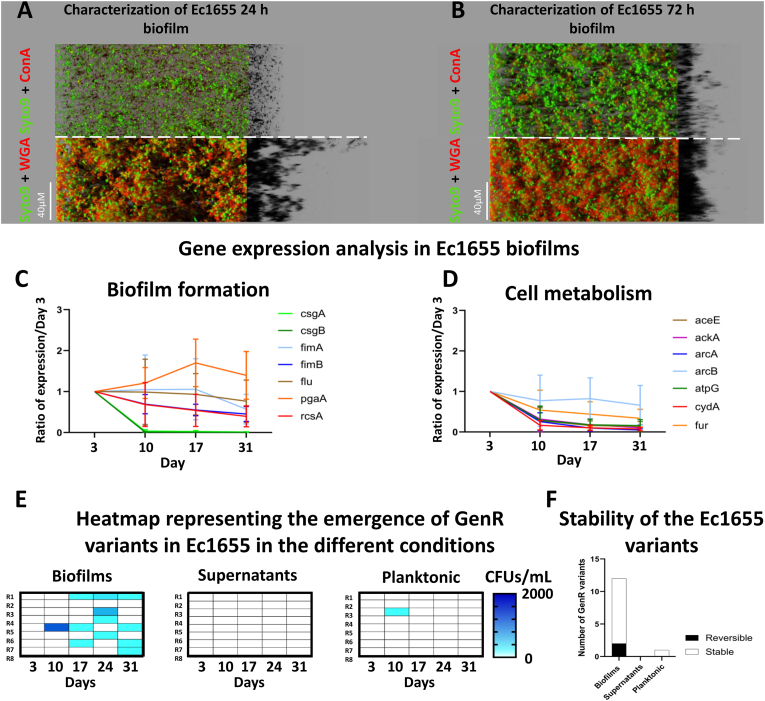
Characterization of the Ec1655 biofilm dynamics and its influence on the emergence of GenR variants. Representative CLSM images of 24 h (A) and 72 h (B) Ec1655 biofilms, stained with Syto9 (green) and ConA (red) in the upper panels, and Syto9 (green) and WGA (red) in the lower panels (four replicates). RT-q-PCR analysis of genes implicated in biofilm formation (C) and cell metabolism (D) in Ec1655 biofilms over a one-month period (three replicates). Quantification of the emergence of Ec1655 GenR variants in biofilms, supernatants and planktonic conditions over a month (E) (eight replicates) and the stability of the resistance phenotype of the emerging variants (F). A Kruskal-wallis test, followed by Dunn's multiple comparison test, was performed on RT-q-PCR data to compare each timepoint. The same tests were used to evaluate the differences in variants emergence between biofilms, supernatants and planktonic cells at each timepoint. P-values are provided in Table S4. (For interpretation of the references to color in this figure legend, the reader is referred to the Web version of this article.)

The biofilm formation of the *E. coli* reference strain Ec1655 was first characterized. ConA staining, which targets polysaccharides containing α-d-mannosyl and α-d-glucosyl residues, showed minimal staining of the biofilm matrix, at both 24 h (Fig. 2A) and 72 h (Fig. 2B). Similarly, a Congo red assay, which detects amyloid fibers and cellulose, confirmed these results in both submerged biofilm (Figs. S1A and S1B) and macrocolony models (Fig. S1C). In contrast, WGA staining, which binds to N-acetylglucosamine residues, revealed well-structured biofilms in which cells were embedded (Fig. 2A and B).

A one-month gene expression analysis of Ec1655 biofilms revealed slight modulations in the expression profiles of genes involved in biofilm formation (Fig. 2C). Notably, *pgaA,* which encodes poly-β-1,6-*N*-acetylglucosamine (PNAG), a key saccharidic component of the extracellular matrix, exhibited a slight increase in expression over the course of the experiment. Conversely, *csgA* and *csgB*, which encode curli proteins involved in adhesion, showed a marked decrease in expression after one week. Given their close link with gentamicin resistance, genes involved in cellular metabolism were also analyzed by RT-q-PCR (Fig. 2D). Interestingly, these genes showed a progressive decline in expression throughout the experiment. Similarly, genes associated with stress responses and membrane transport, both linked to resistance and biofilm metabolism, showed reduced expression levels (Fig. S2). Of the 26 genes analyzed, 17 exhibited significant downregulation between the first and last week of the experiment, including *csgA*, *csgB*, *fimB*, *cpxR*, *dksA*, *hfq*, *recA*, *relA*, *rpoS*, *aceE*, *ackA*, *arcA*, *atpG*, *cydA*, *acrA*, *ompC* and *ompF* (Table S4).

To investigate the emergence of gentamicin-resistant (GenR) variants, biofilms, supernatants and planktonic cultures were sampled weekly over the one-month experimental evolution and plated on gentamicin-supplemented agar (Fig. 2E). A significant difference was observed between biofilm and planktonic lifestyles, with GenR variants predominantly isolated from biofilms. This effect became more pronounced as biofilm maturation progressed, particularly after several weeks of evolution, with statistically significant results at the 24- and 31-day time points (Table S4). No GenR variants were recovered from biofilm supernatants, suggesting that resistance emerged specifically within the 3D-structured biofilm. However, the supernatants contained lower cell densities than the biofilms, which may partly explain the lower frequency of GenR variants (Fig. S3). Finally, an analysis of resistance stability (Fig. 2F) revealed that most isolated GenR variants retained their resistance phenotype after several passages in non-selective conditions, indicating a stable resistance trait.

### 3.2 Strain-dependent diversity in biofilm phenotypes and GenR emergence

**Fig. 3:**
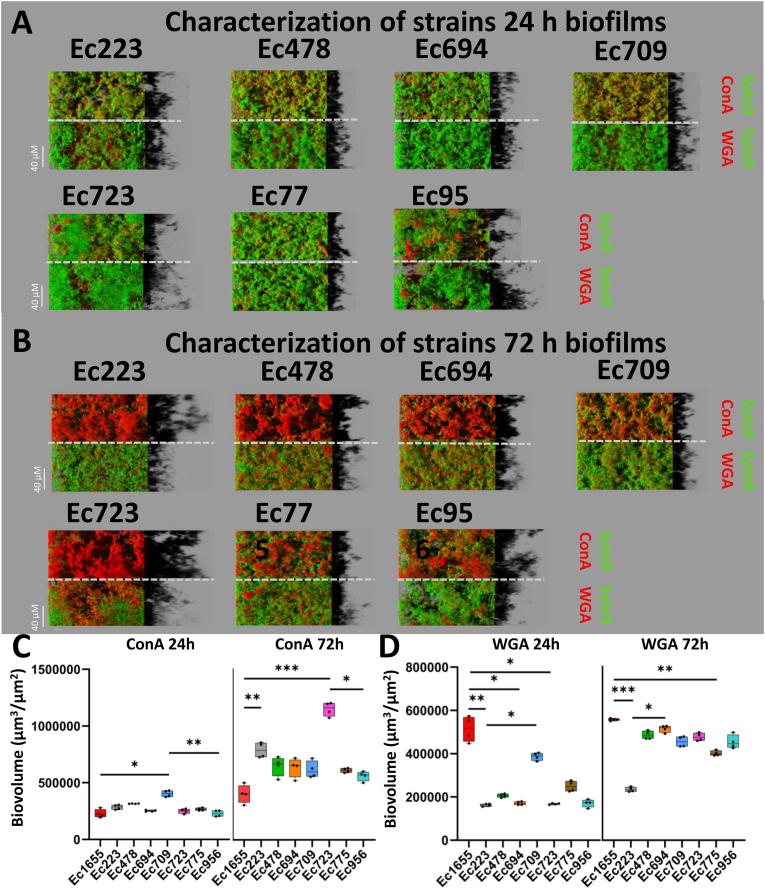
Characterization of the biofilm dynamics of 7 environmental strains and their influence on the emergence of GenR variants. Representative CLSM images of 24 h (A) and 72 h (B) biofilms, stained with Syto9 (green) and ConA (red) in the upper panels, and Syto9 (green) and WGA (red) in the lower panels (four replicates). Quantitative biovolume parameters of the environmental strains are shown, with ConA (C) and WGA (D) staining analyses at both 24 h and 72 h (four replicates). A Kruskal-Wallis test, followed by Dunn's multiple comparison test, was performed to compare quantitative measurements of ConA and WGA across all strains. (P-values: ∗ = [0.05–0.01]; ∗∗ = [0.01–0.001]; ∗∗∗ = <0.001). (For interpretation of the references to color in this figure legend, the reader is referred to the Web version of this article.)

To assess strain-specific variations, the study was extended to seven additional *E. coli* strains isolated from food-processing industries. Biofilm formation was analyzed using CLSM, with 3D-reconstructions displayed in Fig. 3A (24 h) and 3B (72 h). Quantitative structural parameters were extracted from CLSM images (Fig. 3C and D). ConA staining highlighted differences in biofilm matrix composition among environmental strains compared to Ec1655, particularly at 72 h. However, WGA staining revealed that Ec1655 produced more PNAG residues than the other strains (Fig. 3D). Congo red staining (Figs. S1A and S1B) showed that Ec1655 contained fewer amyloid fibers and cellulose than the environmental strains, a finding corroborated by macrocolony analyses (Fig. S1C).

Interesting strain-specific differences emerged from the biofilm analysis. For instance, Ec709 exhibited a strong propensity for early biofilm formation, with significantly higher ConA and WGA signals. At 72 h, Ec723 displayed the most extensive polysaccharidic-rich structures as indicated by ConA staining. Similarly, Ec223 showed high levels of ConA-stained matrix but low WGA-staining, suggesting strain-dependent differences in biofilm composition.

**Fig. 4:**
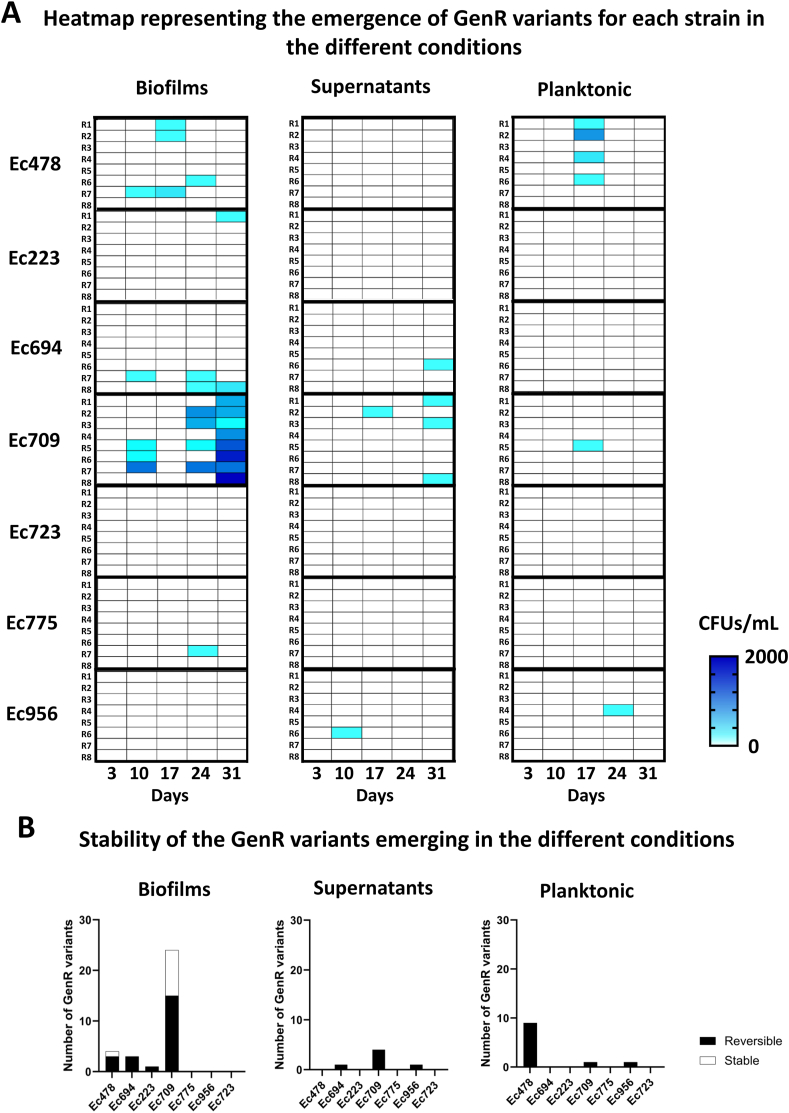
Analysis of the emergence of GenR variants in biofilms, supernatants, and planktonic cells from 7 strains isolated in food-processing industries (A). Stability of the resistance phenotype in the isolated variants (B) (eight replicates). A Kruskal-Wallis test, followed by Dunn's multiple comparison test, was performed to evaluate differences in the emergence of variants between biofilms, supernatants and planktonic cells at each timepoint. P-values are presented in Table S4.

The emergence dynamics of GenR variants were monitored over the one-month experimental period for these strains (Fig. 4A). In Ec709, biofilm growth was associated with a higher frequency of GenR variants compared to supernatants and planktonic cultures. Similar to Ec1655, Ec709 showed a progressive increase in the GenR variant frequency in biofilms over time, with significant differences observed at 24 and 31 days (Table S4). Among the other strains, Ec478 and Ec694 consistently produced GenR variants across multiple replicates and time points. Ec694 produced a majority of variants in biofilms, while Ec478 had variants emerging in both the biofilm and the planktonic conditions, with none detected in biofilm supernatants. As observed with Ec1655, lower cell concentrations in biofilm supernatants may partially explain the absence of GenR variants in this fraction (Fig. S3). In contrast, Ec223, Ec723, Ec775 and Ec956 either did not produce GenR variants or produced them in only one replicate and time point, illustrating strain-dependent variability in evolutionary dynamics. Stability analysis of GenR variants revealed differences compared to Ec1655 (Fig. 4B). In most strains, resistance phenotypes were unstable and lost after passaging on non-selective medium. Notably, Ec709 and Ec478 were the only strains to produce stable GenR variants. All the stable GenR variants were isolated from biofilm conditions, reinforcing the role of biofilms in promoting long-term antibiotic resistance.

### 3.3 Distinct genetic mutations associated with GenR emergence

**Fig. 5:**
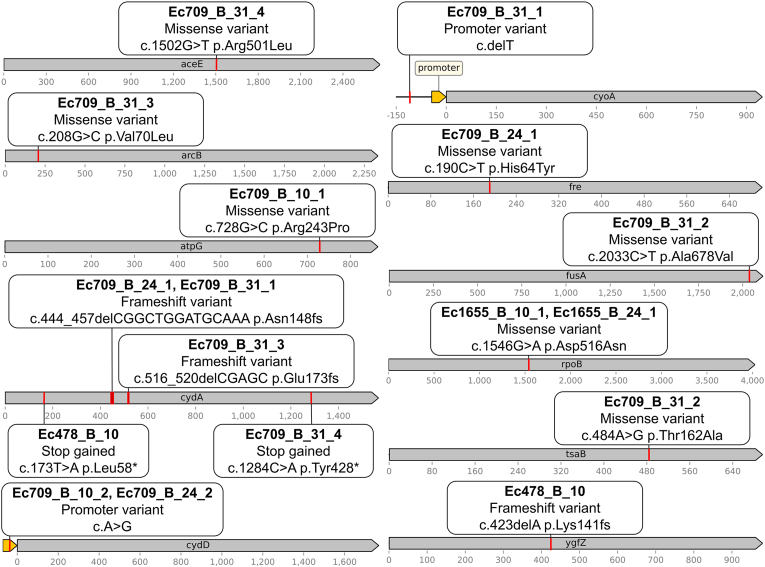
Genetic modifications identified in GenR variants, detailing each mutated gene and its corresponding mutations.

**Fig. 6:**
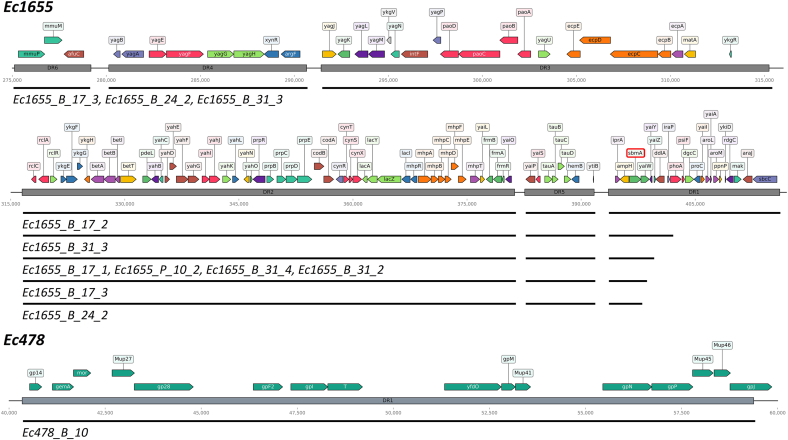
Large genomic modifications identified in GenR variants, along with the corresponding deleted regions (DR).

Genetic analysis of the stable GenR variants revealed distinct mutational profiles between the food isolates and the reference strain Ec1655 (Fig. 5, Fig. 6). The Ec478 and Ec709 GenR variants frequently carried point mutations in genes involved in the respiratory chain (Fig. 5), particularly in *cydA*, which encodes a cytochrome oxidase. This gene was mutated in the Ec478 variant and in four out of nine Ec709 variants. Additionally, some Ec709 variants carried a mutation in an intergenic region between *trxB* and *cydD,* with further analysis revealing that these mutations were located between the *cydCD* operon promoter and the *cydD* gene, potentially affecting gene regulation. Most Ec478 and Ec709 variants also carried a second mutation in another gene with metabolic properties. Specifically, the Ec478 variant exhibited a mutation in the folate-binding protein *ygfZ*, while the *cydA*-mutated Ec709 variants harbored mutations in the flavin reductase *fre*, in an intergenic region between *ampG* and *cyoA*, in the cell respiration regulator *arcB* and the pyruvate dehydrogenase *aceE.* Additionally, two other Ec709 variants did not carry mutations in *cydA,* but instead in other genes. Ec709_B_10_1 was mutated in the ATP synthase gene *atpG,* and Ec709_B_31_2 was mutated in the elongation factor *fusA* and the threonylcarbamoyladenosine synthase *tsaB*. One variant, Ec709_B_24_3 did not carry any point mutation in any gene.

These genetic profiles were markedly different from those observed in Ec1655 GenR variants. Among the ten Ec1655 GenR variants analyzed, only two displayed point mutations, located in the gene *rpoB,* which encodes an RNA polymerase subunit (Fig. 5). The remaining eight variants, including Ec1655_P_10_2 (the sole variant emerging under planktonic conditions), exhibited large genomic deletions instead of point mutations (Fig. 6). Interestingly, while these deletions occurred within the same genomic region, their sizes varied across the different variants, suggesting independent deletion events. Among the genes found in the deleted region, the peptide transporter *sbmA* was deleted in every strain, and is known to be involved in gentamicin resistance [15,30]. Other genes in the deleted region were mainly involved in diverse metabolic processes, including *iprA*, whose deletion confers high resistance to oxidative stress [31]. Three strains (Ec1655_B_17_3, Ec1655_B_24_2, Ec1655_B_31_3) exhibited even larger deletions extending upstream of the *rclC* gene, leading to the loss of genes encoding ferric transporters, fimbriae and other metabolism-related proteins. Interestingly, the deleted region in Ec1655 variants contained few mobile genetic elements. In contrast, analysis using the MobileOG software (Fig. 6) revealed that most of the deleted region in the Ec478 variant consisted of phage-related genes. No large deletions were observed in Ec709 variants.

### 3.4 Broad-spectrum aminoglycoside resistance in GenR variants

The resistances profiles of the GenR variants against 15 antibiotics relevant to *E. coli* were assessed by determining their minimum inhibitory concentration (MIC) values using the microdilution broth method. This analysis aimed to evaluate whether the resistance mutations conferred cross-resistance to other antibiotics (Table 1). Among the tested antibiotics, two belonged to the aminoglycoside family: gentamicin and amikacin. A decrease in susceptibility to gentamicin was observed in all the variants. Moreover, the MIC values for amikacin confirmed that the resistance in the GenR variants extended to other aminoglycosides. Interestingly, Ec1655 and most of the Ec709 variants exhibited decreased susceptibility to sulfamethoxazole and trimethoprim, while the Ec478 variant remained unaffected. In terms of resistance levels, Ec1655 GenR variants were generally resistant to higher concentrations compared to Ec709 and Ec478 variants. Interestingly, a majority of Ec1655 and Ec478 variants displayed slightly increased sensitivity to ampicillin, suggesting that the resistance mutations might have pleiotropic effects on other antibiotics.

**Table 1:**
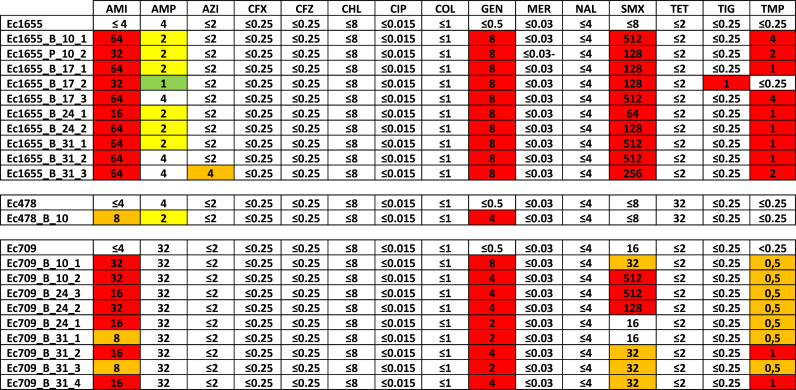
Resistance to 15 antibiotics was assessed for each variant and compared to the resistance profiles of their parental strains. Changes in resistance levels are color-coded as follows: more than a two-fold increase in resistance is shown in red, a two-fold increase in orange, a two-fold decrease in yellow, and more than a two-fold decrease in green. (AMI = Amikacin; AMP = Ampicillin; AZI = Azithromycin; CFX = Cefotaxime; CFZ = Ceftazidime; CHL = Chloramphenicol; CIP = Ciprofloxacin; COL = Colistin; GEN = Gentamicin; MER = Meropenem; NAL = Nalidixic Acid; SMX = Sulfamethoxazole; TET = Tetracycline; TIG = Tigecycline; TMP = Trimethoprim).

### 3.5 Variants exhibit altered recolonization properties

**Fig. 7:**
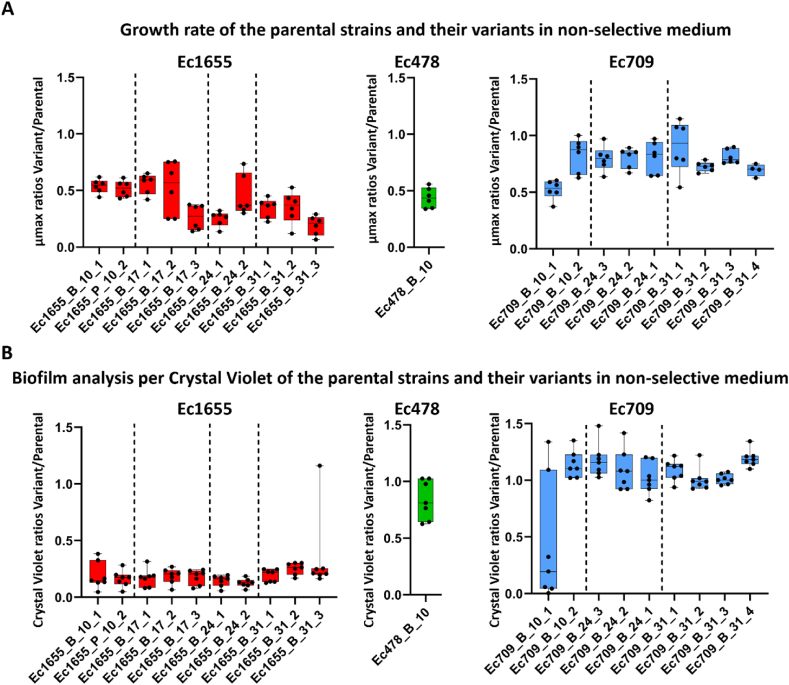
Graph representing the ratio of variants compared to their parental strain, for their maximum growth rate (A) and biofilm formation ability per Crystal Violet assay (B) (six replicates). Dashed lines separate the different sampling days of the variants. Mann-Whitney test was performed for each variant versus its corresponding parental strain. P-values are provided in Table S4. (For interpretation of the references to color in this figure legend, the reader is referred to the Web version of this article.)

Phenotypic analyses were carried out to assess the fitness trade-offs and biofilm formation abilities of the GenR variants, providing insight into their capacity to disseminate and colonize new environments (Fig. 7). Growth kinetics were performed in the absence of selective pressure, and the maximal growth rates (μmax) were compared between the GenR variants and their parental strains (Fig. 7A). In general, GenR variants showed decreased growth rates compared to their ancestors, but with disparities between strains. Ec1655 variants were particularly affected, with no notable differences between the variants carrying point mutations in *rpoB* and those with large genomic deletions. Ec478_B_10 was also severely affected. In contrast, most of the Ec709 GenR variants displayed moderate reductions in their growth rates, with some variants showing growth rates comparable to their parental strain.

Crystal Violet biofilm quantification (Fig. 7B) revealed that Ec1655 GenR variants were unable to produce biofilms, in contrast to their parental strain. Conversely, Ec478 and Ec709 GenR variants produced similar biofilm biomass as their respective parental strains.

## 4 Discussion

Bacterial adaptation is influenced by multiple factors, with biofilms playing a crucial role in microbial community evolution. As the predominant bacterial lifestyle on Earth [32], understanding how biofilms contribute to the emergence of antibiotic resistances is essential for addressing this significant public health issue. In this study, we explored the dynamics of GenR variants emergence in biofilms, supernatants and planktonic conditions using the *E. coli* laboratory reference strain Ec1655 and seven food-processing isolates.

Our findings revealed that the emergence of the GenR phenotype was privileged in biofilms, compared to supernatants and planktonic cultures, particularly after three to four weeks of evolution. Resistance stability assays (Fig. 4B) showed that a significant number of GenR phenotypes in various strains relied on adaptive resistance, which was lost after successive passages on non-selective medium. Biofilm maturation and resilience to antimicrobial treatments are often linked to the formation of dormancy-like subpopulations with reduced cellular activity [33]. Dormant cells evade antimicrobials action by lowering the activity of drug targets [34]. Gentamicin, which requires active cellular metabolism to exert its antimicrobial effect, is less effective against cells with reduced metabolic activity [14,16,35]. This is consistent with the gradual decrease in the expression of metabolic genes observed in Ec1655 biofilms (Fig. 2D). Interestingly, the expression of the *pgaA* gene, which encodes a saccharidic matrix component, increased slightly during the experiment, suggesting a role in the development and shaping of the mature biofilm structure. Biofilm maturation generates chemical gradients through the establishment of a three-dimensional structure, influencing bacterial physiology [6].

In addition to metabolic shifts, the settlement of specific microenvironments within biofilms could promote genetic diversity. This aligns with the observation that a vast majority of the GenR variants with stable resistance phenotypes due to genetic mutations arose preferentially in biofilm conditions. Harsh biofilm-associated conditions are known to potentiate genetic diversification, with variants being selected within the protective structure of the biofilm [9,12,36]. However, no direct correlation between matrix production capacity and diversity generation was observed in this study, supporting that additional strain-specific factors likely contribute to GenR emergence in biofilms. Indeed, biofilm matrix abundance was mostly similar across strains in 72h biofilms. Moreover, while gene expression analysis also showed a decrease in central metabolism activity during Ec223 biofilm maturation (Fig. S4), this strain did not produce any GenR variants. These observations underscore the complex relationship between biofilm biovolume matrix production, cellular metabolism and resistance emergence.

Here, the strain-specific emergence of GenR variants is likely influenced by genetic background. *E. coli* exhibits substantial intra-species diversity [37] and a strain's genetic makeup significantly influences its evolutionary adaptation. Strains that successfully adapt and survive are not necessarily those with the highest initial fitness [38,39]. Interactions among background genes, their targets and other genetic factors can determine whether a mutation is beneficial and selected for [40,41]. Here, Ec709 was phylogenetically distant from the other strains, belonging to the ST131 sequence type, which is implicated in extra-intestinal infections, particularly urinary tract and bloodstream infections [42]. Genomic comparisons between Ec709 and the other parental strains revealed 115 genes unique to the Ec709 genome. Among these genes, only 15 had an identified function, often linked to ATP production (data not shown).

Genomic analyses reflected distinct adaptation strategies for Ec1655, in contrast to the ones observed in Ec709 and Ec478. Variants of Ec709 and Ec478 predominantly exhibited SNPs or small indels in genes already associated with bacterial resistance to gentamicin. These targets were particularly related to cell respiration, such as *cydA*, *cydD*, *atpG*, *arcB* and *cyoA* [14,21,35]. Additional mutations in genes like *fusA* [15,35] and *aceE* [17] were also directly linked to gentamicin resistance in *E. coli*. In contrast, GenR variants derived from Ec1655 were characterized by their large genomic deletion rather that point mutations. Among this deletion, *sbmA,* a gene encoding a peptide transporter involved in gentamicin internalization [30], was systematically lost. The loss of *sbmA* has been previously associated with reduced bacterial sensitivity to aminoglycosides in *E. coli* [43]. This deletion could participate here to the increase of Ec1655 aminoglycoside resistance. Notably, similar resistance mechanisms have already been documented in *E. coli* biofilms, where early mutations in *sbmA* emerge in the presence of amikacin [15]. The varying size of these deletions suggest they arose from independent genetic events. Additionally, analysis of mobile genetic elements presence in the region revealed that, unlike the deletion observed in Ec478_B_10 (likely due to phage excision), the affected region in Ec1655 should have been stably anchored in the genome.

Further analysis of the deleted region revealed numerous genes involved in diverse metabolic functions. Among them, *iprA* gene was also consistently deleted in Ec1655 GenR variants. Previous studies have linked *iprA* deletions to enhanced resistance to oxidative stresses [31], which are commonly found in biofilm matrices [44]. This suggests that the deletion of this region could represent an adaptation to the biofilm lifestyle, offering protection against oxidative stresses and concomitantly reducing cellular activity. The proximity of *sbmA* to these genes implies that its deletion—and the resulting gentamicin resistance—may have occurred as a side effect of this broader adaptive mechanism. Phenotypic analyses confirmed severe fitness cost in Ec1655 variants, including significant reductions in growth rates and biofilm production. Such growth defects are consistent with the known fitness costs of aminoglycoside resistances in *E. coli* [45]. While all variants demonstrated decreased susceptibility to aminoglycosides, with impacts on amikacin and gentamicin resistance, Ec1655 variants displayed higher MIC values compared to those of Ec478 and Ec709. This aligns with the established correlation that higher resistance levels have greater fitness costs [11]. These findings support the hypothesis that Ec1655 variants, while capable of surviving within biofilm microenvironments, were too severely impaired to compete in a planktonic lifestyle. In contrast, Ec709 and Ec478 variants, despite reduced growth rates, maintained relative fitness levels and retained biofilm formation capacity. This supports a metabolic reorientation favoring biofilm production, allowing these variants to sustain biofilm biomass despite growth impairments. These observations highlight the potential of GenR variants originating from food-processing isolates to recolonize and disseminate on surfaces, representing a possible food safety risk within a One Health perspective.

## 5 Conclusion

This study investigated the mechanisms driving the selection of GenR variants in *E. coli* populations, highlighting the critical role of biofilm lifestyles in fostering genetic diversity. Our findings underscore the complexity of this selection process, which is highly strain-dependent and shaped by both genetic and environmental factors. The study offers valuable insights into how biofilm development contributes to the emergence of antimicrobial resistance, carrying significant public health implications. By deepening our understanding of these dynamics, this work lays the groundwork for designing more effective interventions to combat biofilm-associated infections and curb the spread of resistance.

## CRediT authorship contribution statement

**Raphaël Charron:** Writing – review & editing, Writing – original draft, Visualization, Investigation, Formal analysis, Conceptualization. **Pierre Lemée:** Writing – original draft, Software, Methodology, Investigation, Formal analysis, Data curation. **Antoine Huguet:** Investigation, Formal analysis. **Ornella Minlong:** Investigation. **Marine Boulanger:** Investigation. **Paméla Houée:** Investigation. **Christophe Soumet:** Resources, Project administration. **Romain Briandet:** Writing – review & editing, Writing – original draft, Supervision, Funding acquisition, Conceptualization. **Arnaud Bridier:** Writing – review & editing, Writing – original draft, Visualization, Supervision, Resources, Project administration, Funding acquisition, Conceptualization.

## Data availability

Detailed accession numbers for assembled genomes of parental strains and sequence reads of variant strains were provided in Table S3.

## Declaration of competing interest

The authors declare that the research was conducted in the absence of any commercial or financial relationships that could be construed as a potential conflict of interest. Given his role as Editor, Romain Briandet had no involvement in the peer review of this article and has no access to information regarding its peer review. Full responsibility for the editorial process for this article was delegated to Tom Coenye.
