# Supplementary material for: Strain-dependent emergence of aminoglycoside resistance in Escherichia coli biofilms
Source: Biofilm. 2025 Mar 12;9:100273. doi: 10.1016/j.bioflm.2025.100273 (PMC11952850; doi:10.1016/j.bioflm.2025.100273)
Supplement: Multimedia component 1 [file mmc1.docx]

**Supplementary Data:**

**Supplementary Material and Methods:**

**1. Congo red fluorescence assay**

Biofilms of the parental strains were prepared at 24 h and 72 h as described in section 2 of the main Material and Methods. A solution of 50 µL of TSB 1/10 supplemented with Congo red (Sigma-Aldrich, France) was added to each well to achieve a final concentration of 40 µg/mL. The plates were incubated in the dark for 15 minutes. Fluorescence was quantified using a Biotek SynergyH1 microplate reader, with excitation at 525 nm and emission at 625 nm. Three biological replicates with three technical replicates were performed for each strain under each condition.

**2. Macrocolony observation**

Strains were streaked on TSA plates and incubated overnight at 37 °C. On the following day, a colony from each strain was suspended in TSB 1/10 and grown overnight at 37 °C. The next day, cultures were diluted to achieve a final OD_600 nm_ of 0.2. 10 µL of the diluted suspensions were dropped onto TSA plates supplemented with 40 µg/mL Congo red and 20 µg/mL brilliant blue. Plates were incubated in the dark at 19°C or 37 °C for 7 days, after which they were photographed using a Canon EOS R10 camera.

**3. Colony forming unit (CFUs) quantification**

Cells from 72 h biofilms, their supernatants and planktonic suspensions were counted to observe any differences in cell quantities and densities between the conditions in the different parental strains. 72 h biofilms were prepared as described in section 2 of the main material and methods, and planktonic cultures as in section 3. 20 µL of the planktonic cultures were suspended in 180 µL of TSB 1/10 medium. Serial dilutions were performed to achieve a 10^-8^ dilution. 10 µL of each dilution was dropped onto TSA plates. The same steps were repeated for the supernatants. For the biofilms, the supernatants were first removed and replaced with 100 µL of fresh TSB 1/10 medium. The biofilms were scratched and resuspended. The 100 µL suspension was transferred to 900 µL of TSB 1/10 medium. Tubes were vortexed thoroughly during 30 s to separate the biofilm aggregates. Serial dilutions were then performed using 20 µL suspensions in 180 µL TSB 1/10 medium, and 10 µL of the dilutions were dropped onto TSB 1/10 medium. Finally, plates were incubated overnight at 37 °C and counted the following day. Three biological replicates were performed for each strain.

**Figure S1:**


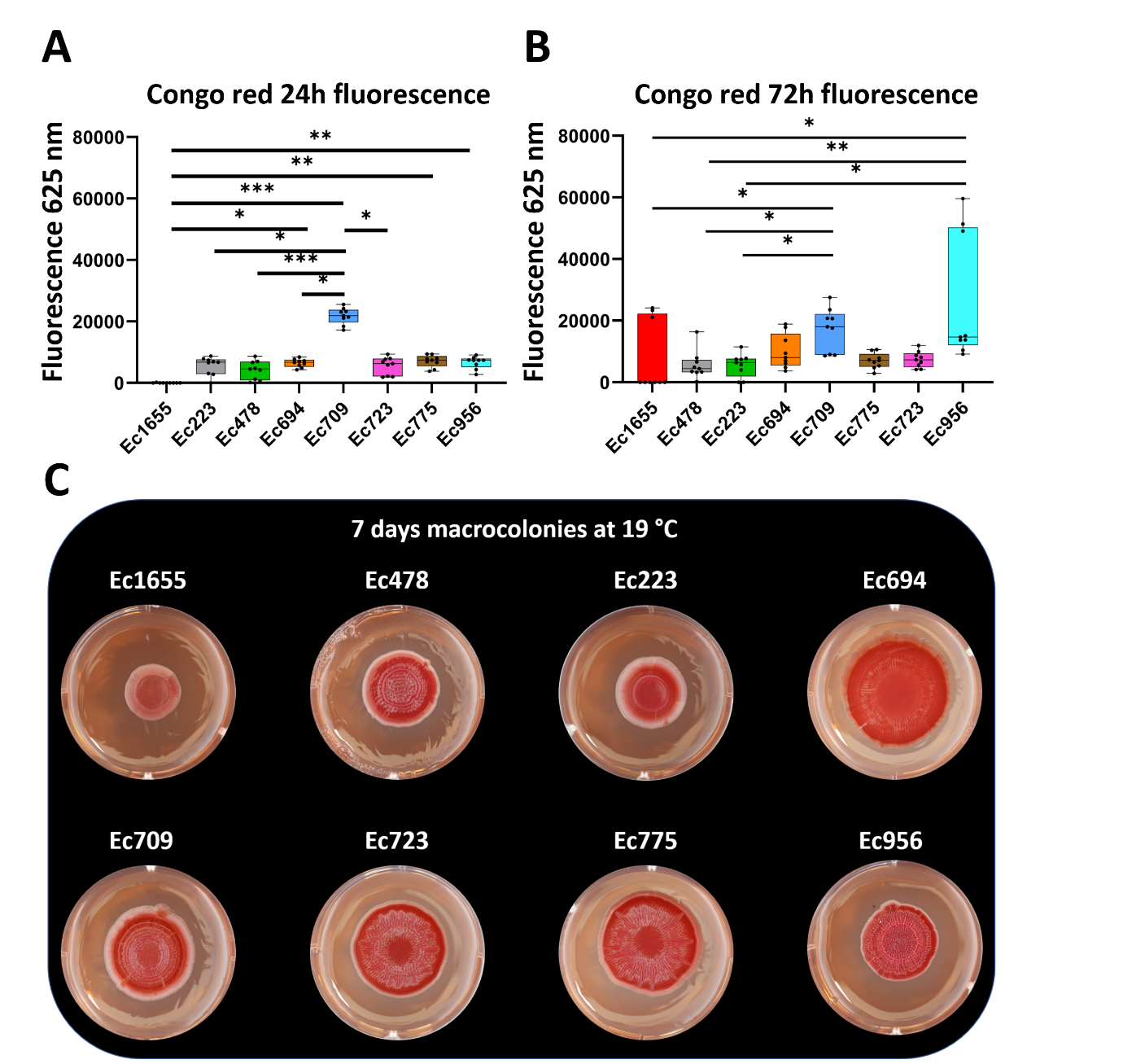


*Figure S1: Congo red fluorescence values in submerged biofilms of the different strains at 24h (A) and 72h (B) (nine replicates). Macrocolonies of the different strains, stained with Congo red and brilliant blue (C). A Kruskal-Wallis test, followed by Dunn’s multiple comparison test, was performed on the Congo red fluorescence values (P value: * = [0.05-0.01]; ** = [0.01-0.001]; *** = <0.001)*

**Figure S2:**

**
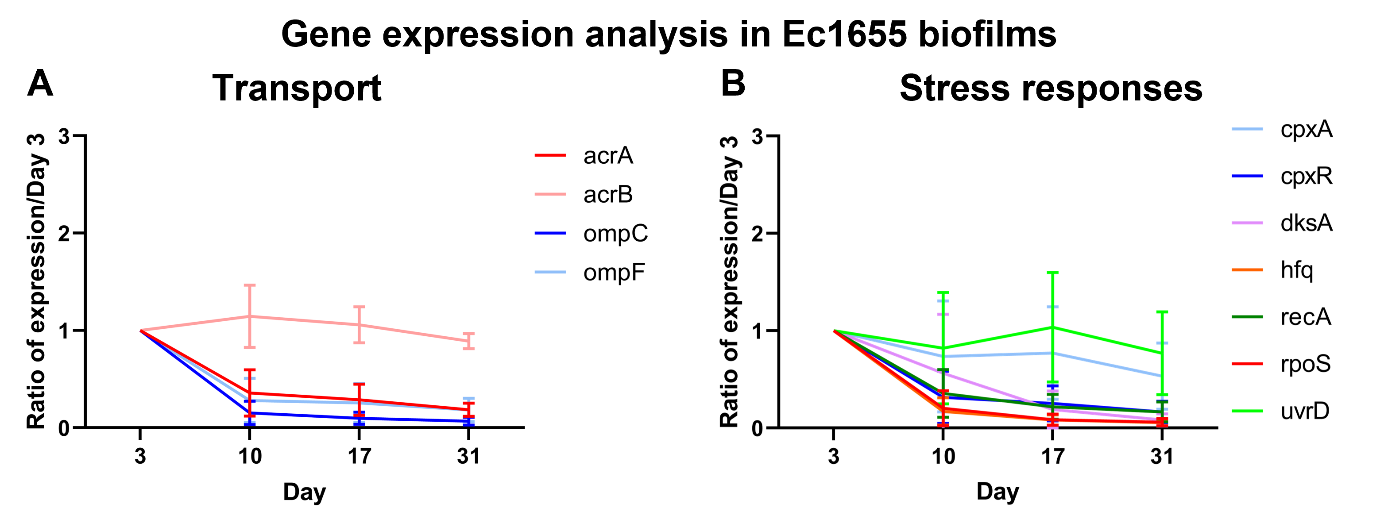
**

*Figure S2: Gene expression levels in Ec1655 biofilms for genes implicated in transport (A) and stress responses (B) (three replicates). A Kruskal-wallis test, followed by Dunn’s multiple comparison test, was performed on RT-q-PCR values to compare each timepoint. P-values are provided in Table S4.*

**Figure S3:**

*
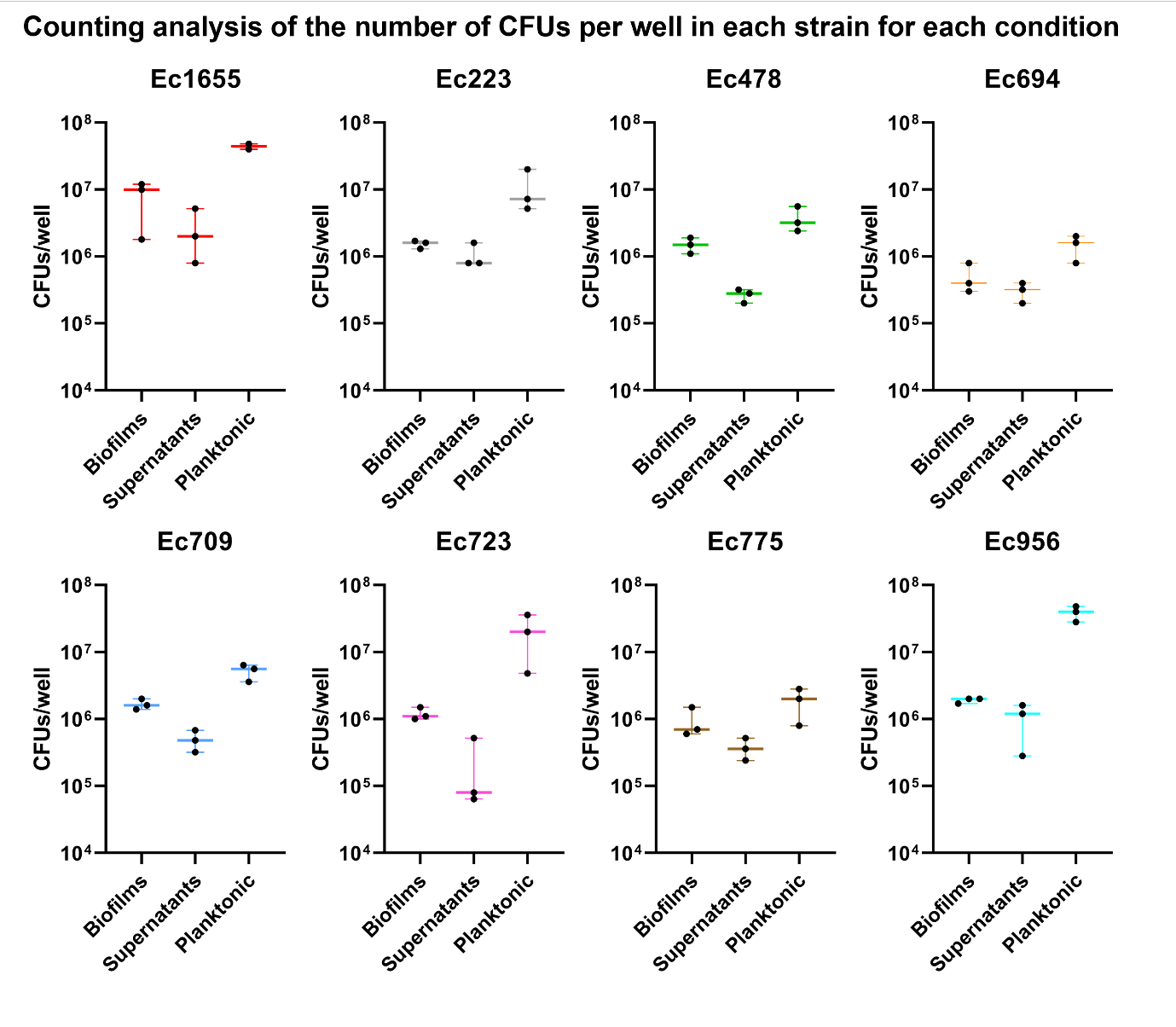
Figure S3: Number of CFUs/well in biofilms, supernatants and planktonic cultures, at 72 h, for each strain (three replicates).*

**Figure S4:**

**
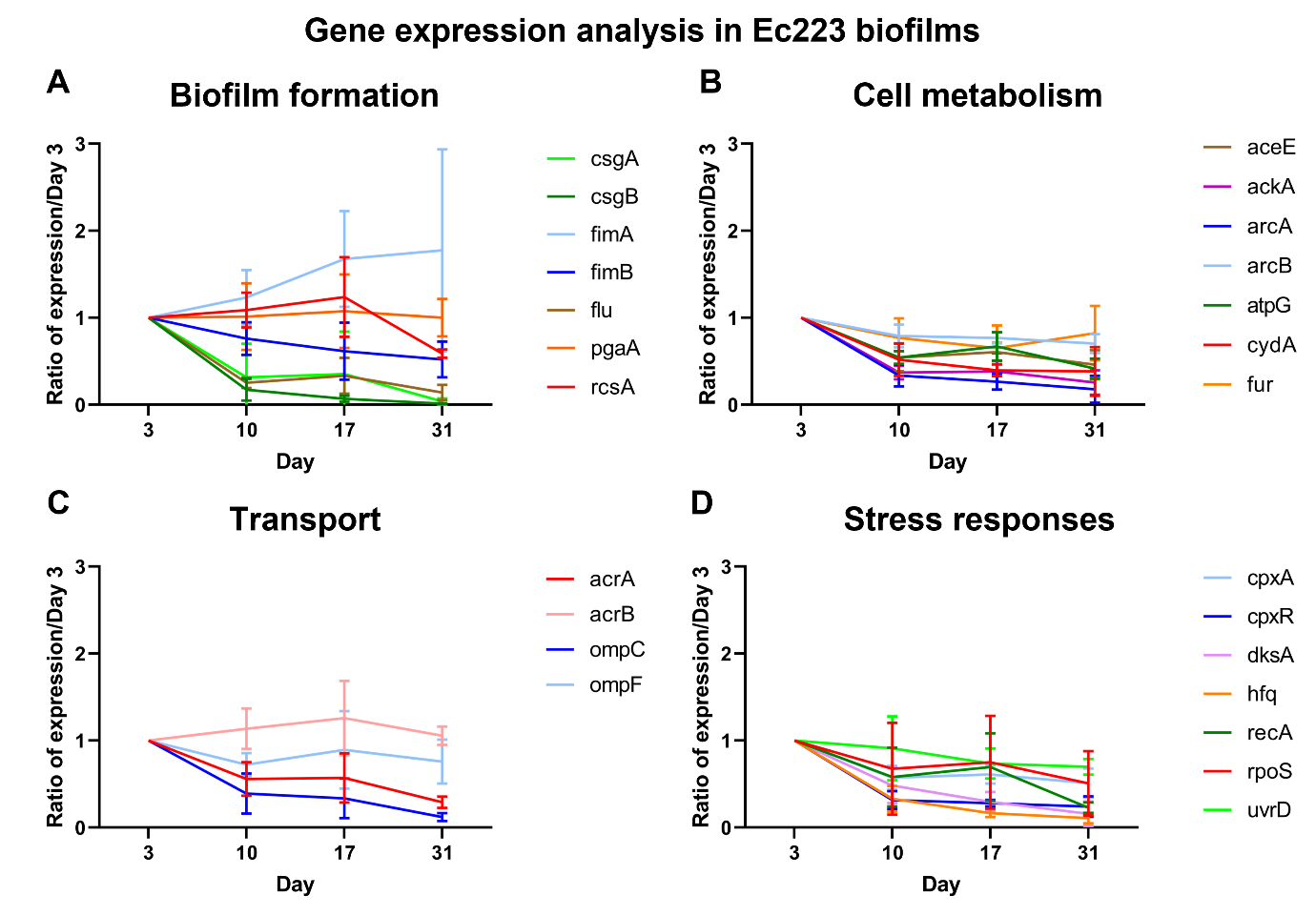
**

*Figure S4: One-month gene expression analysis in Ec223 biofilms for genes implicated in biofilm formation (A), cell metabolism (B), transport (C) and stress responses (D) (three relicates). A Kruskal-wallis test, followed by Dunn’s multiple comparison test, was performed on RT-q-PCR values to compare each timepoint. P-values are available in Table S4.*

Table S1. Target genes and primers used in this study

Table S2: Detailed information on the genetic modifications

Table S3: NCBI accession number for every sequenced genome

Table S4: P-values obtained with the different statistical analyses performed on the experiments throughout the study
